# Central nervous system-related safety and tolerability of add-on ketamine to standard of care treatment in treatment-resistant psychotic depression in patients with major depressive disorder and bipolar disorder

**DOI:** 10.3389/fnins.2023.1214972

**Published:** 2023-07-11

**Authors:** Maria Gałuszko-Wȩgielnik, Katarzyna Jakuszkowiak-Wojten, Mariusz Stanisław Wiglusz, Wiesław Jerzy Cubała, Michał Pastuszak

**Affiliations:** Department of Psychiatry, Faculty of Medicine, Medical University of Gdańsk, Gdańsk, Poland

**Keywords:** ketamine, bipolar depression, psychotic depression, treatment-resistant depression, dissociation

## Abstract

**Background:**

Psychotic treatment-resistant depression represents a complex and challenging form of mood disorder in clinical practice. Despite its severity, psychotic depression is frequently underdiagnosed and inadequately treated. Ketamine has demonstrated rapid and potent antidepressant effects in clinical studies, while exhibiting a favorable safety and tolerability profile. Although there is limited literature available on the use of ketamine in psychotic TRD, reports on its efficacy, safety, and tolerability profile are of great interest to clinicians. The aim of this study is to investigate the relationship between dissociative symptomatology and psychomimetic effects in inpatients with treatment-resistant major psychotic depression and treatment-resistant bipolar psychotic depression, who receive intravenous ketamine treatment alongside psychotropic medication, both during and after treatment.

**Materials and methods:**

A total of 36 patients diagnosed with treatment-resistant unipolar (17 patients) or bipolar (18 patients) depression with psychotic features were treated with eight intravenous infusions of 0.5 mg/kg ketamine twice a week over 4 weeks. Ketamine was given in addition to their standard of care treatment. The severity of depressive symptoms was evaluated using the MADRS, while dissociative and psychomimetic symptoms were assessed using the CADSS and BPRS, respectively.

**Results:**

There were no statistically significant changes observed in MADRS, CADSS, and BPRS scores within the study group during ketamine infusions. However, significant improvements in MADRS, CADSS, and BPRS scores were observed during ketamine infusions in both the unipolar and bipolar depression groups.

**Conclusion:**

This study provides support for the lack of exacerbation of psychotic symptoms in both unipolar and bipolar depression.

## 1. Introduction

Initially it was believed that psychotic depression was located at one end of a spectrum of major depression severity. However, subsequent research has demonstrated that psychosis is a completely distinct characteristic that may co-occur with varying degrees of mood disorders (Dubovsky et al., 2021). Compared to non-psychotic depression, psychotic depression is more likely to have a bipolar outcome and episodes of bipolar depression are more commonly associated with psychotic symptoms compared to episodes of unipolar depression (Guze et al., 1975; Goodwin and Jamison, 1991; Østergaard et al., 2015). Bipolarity is a strong predictor of psychosis in the course of a mood disorder (Souery et al., 2011). Nonetheless, the presence of psychosis indicates a more severe primary disorder that causes more impairment and has a worse prognosis (Jääskeläinen et al., 2018). It is worth noting that spontaneous recovery rates for psychotic depression are low (Glassman and Roose, 1981).

In the treatment of psychotic unipolar depression, the current standard involves administering a combination of an antidepressant and an antipsychotic medication, or utilizing electroconvulsive therapy (Leadholm et al., 2013). However, there is insufficient data available regarding the maintenance treatment of unipolar psychotic depression, as well as the acute and long-term treatment of psychotic bipolar disorder. As a result, medical professionals must still rely on clinical experience to make informed treatment decisions.

Medical evidence strongly supports the use of ketamine as a means of quickly alleviating symptoms in patients with treatment-resistant depression (TRD) who suffer from major depressive disorder (MDD) or bipolar disorder (BD) (Kryst et al., 2020; McIntyre et al., 2020; Wilkowska et al., 2021; Papp et al., 2022). However, due to the potential risks of psychomimetic and dissociative effects (Short et al., 2018), most ketamine studies have excluded patients with psychosis due to concerns that it could worsen symptoms (Beck et al., 2020). Nevertheless, several studies have demonstrated that ketamine not only improves mood, but also reduces psychotic symptoms in patients with TRD who experience psychosis. The treatment shows good safety and tolerability (da Frota Ribeiro et al., 2016; Pennybaker et al., 2017; Ajub and Lacerda, 2018; Kim et al., 2021; Veraart et al., 2021; Gałuszko-Wȩgielnik et al., 2023).

In medical terms, apprehensions regarding the administration of ketamine to individuals with a history of psychosis can be traced back to the 1990s when ketamine was utilized in schizophrenia research (Krystal et al., 1994). Later studies erroneously labeled expansive or mystical-type experiences reported by subjects receiving ketamine for depression as “psychotomimetic” (Zarate et al., 2006). This misinterpretation resulted in some practitioners excluding patients with treatment-resistant bipolar depression from receiving ketamine therapy. However, this exclusion may not be justified if patients are stabilized on medication (Bennett et al., 2022).

One of the primary concerns revolves around the potential occurrence of adverse events linked to dissociative symptomatology (Włodarczyk et al., 2019). While there is limited evidence available from a small number of studies (Correia-Melo et al., 2017), suggesting that dissociative symptoms may serve as predictors of response in treatment-resistant depression (TRD) encompassing both major depressive disorder (TRD-MDD) and bipolar disorder (TRD-BP), the understanding of this relationship remains inadequate. Additionally, there is a scarcity of data pertaining to dissociative symptomatology in individuals receiving ketamine treatment for depression (McCloud et al., 2015). However, some indications from studies on esketamine (Daly et al., 2019; Fedgchin et al., 2019; Popova et al., 2019) suggest that dissociative symptoms tend to diminish over time, indicating a potential long-term symptom profile. The hypothesis posits an association between dissociation and treatment outcomes, yet the existing body of evidence does not strongly support this finding (Mathai et al., 2023).

Recently, several studies have been monitoring mystical-type experiences as an indicator of clinical improvement. Mystical experiences, characterized by features such as oceanic boundlessness, ego dissolution, universal interconnectedness, and transcendence of time and space, have been identified as a potential psychological mechanism. A systematic review of 12 studies examined the use of psychedelics in adults with psychiatric disorders, including substance use disorder, depressive disorders, and cancer-related distress. The review proposed that the presence and intensity of mystical experiences may contribute to therapeutic efficacy, resulting in symptom reduction and improved quality of life. Future research should consider a larger and more diverse samples using randomized designs to further investigate this phenomenon (Ko et al., 2022).

The objective of this study is to examine the correlation between dissociative symptomatology and psychomimetic effects (psychotic symptoms) in inpatients with treatment-resistant major psychotic depression (TRD-MDD-P) and treatment-resistant bipolar psychotic depression (TRD-BD-P) who undergo intravenous ketamine treatment in conjunction with psychotropic medication, both during and after treatment.

## 2. Materials and methods

Participants were enrolled in a naturalistic observational registry strategy to assess the safety and tolerability of ketamine infusions in TRD (NCT04226963). Included were inpatients with a TRD with psychotic characteristics in the course of severe depression (17 individuals) or bipolar disorder (18 subjects). Clinicians investigated the subjects using the Mini-International Neuropsychiatric Interview to confirm the diagnosis based on the Diagnostic and Statistical Manual of Mental Disorders criteria (DSM 5). All the subjects demonstrated resistance to treatment for the present episode, which was defined as an unsatisfactory response to two appropriate and sufficient treatment interventions according to clinical standards (Poon et al., 2015). The research used a single patient and a single rater. The physician graded patients using the Montgomery Asberg Depression Rating Scale (MADRS), Young Mania Rating Scale (YMRS), Columbia–Suicide Severity Rating Scale (CSSRS), the Clinician Administered Dissociative Symptoms Scale (CADSS), and Brief Psychiatric Rating Scale (BPRS) throughout the screening procedure. The CADSS was used since it is the most often used instrument for assessing the acute psychoactive effects of ketamine administration in previous studies for mood disorders (Bremner et al., 1998), and the BPRS with the 4-item positive symptom subscale was selected as a safety evaluation (Włodarczyk and Cubała, 2020). Adult inpatients between 18 and 65 years old who were medically stable, communicative, and able to give informed consent were the only participants recruited in the research. During therapy with ketamine, several patients with severe somatic illnesses continued to use their present medications. Exclusion criteria included a history of uncontrolled medical disorders, a previous bad response to ketamine, pregnancy, or breastfeeding. All individuals provided given written permission to participate in the research. The research was conducted in compliance with the most recent version of the Helsinki Declaration. After thoroughly explaining the methods each participant signed informed consent. The Independent Bioethics Committee for Scientific Research at Medical University of Gdańsk, Poland, accepted the research protocols: NKBBN/172/2017; 172-674/2019.

### 2.1. Ketamine infusions

During ketamine infusions, all patients continued conventional psychotropic and chronic somatic illness therapy. Over 4 weeks, eight ketamine infusions were administered as the study’s therapeutic intervention. All infusions of ketamine were administered intravenously over 40 min at a rate of 0.5 mg/kg depending on the patient’s actual body weight. The attending psychiatrist monitored the patient’s safety before, throughout, and up to an hour and a half after the infusion, every 15 min. The procedure comprised of regular monitoring of vital signs such as heart rate, body temperature, respiratory rate, blood pressure, and oxygen saturation, as well as a mental status examination that involved evaluating the presence of psychotic and dissociative symptoms using BPRS and CADSS before and 30 min after the infusion. Before the 1st, 3rd, 5th, and 7th infusions and during the follow-up (1 week after 8th infusion), psychometric assessment with the YMRS and MADRS was conducted. The ECG was performed before every other infusion and 1 week after the last ketamine administration. All physicians were licensed psychiatrists, knowledgeable with the behavioral therapy of patients with significant mental status changes, and prepared to handle any emergency behavioral issues. Additionally, a physician on-site examined the patient for possible behavioral concerns, such as suicidal thoughts, after each session. A participant was considered as a responder if their MADRS total score improved by at least 50 percent (comparing the follow up visit with baseline). A patient was supposed to be remitter if their total MADRS score was ≤10 points (measured in follow up visit) (Trivedi et al., 2009).

## 3. Statistical analysis

All statistical calculations were conducted using StatSoft. Inc. (2014). STATISTICA (data analysis software system), version 12.0. www.statsoft.com, and Microsoft Excel spreadsheet.

Continuous variables were characterized using mean, standard deviation (SD), median, minimum and maximum values (range), and 95% confidence intervals (CI). Categorical variables were presented as frequencies and percentages. To determine whether a continuous variable was normally distributed, the Shapiro–Wilk test was used. The Levene test (or Brown–Forsythe’s test) was used to test the hypothesis of equal variances. To examine the significance of differences between two groups (unpaired variable model), significance tests, including the Student’s *t*-test (or Welch’s test in case of unequal variances), or the Mann–Whitney U test (for ordinal data or non-normally distributed data) were used. The significance of differences between more than two groups was evaluated using the F test (ANOVA) or the Kruskal–Wallis test (for non-normally distributed data or ordinal data). In case of obtaining statistically significant differences between groups, *post-hoc* tests were applied, including Tukey’s test for ANOVA and Dunna’s test for Kruskal–Wallis. For the paired variable model, the Student’s *t*-test or Wilcoxon signed-rank test (for ordinal data or non-normally distributed data) was used. The significance of differences between more than two groups in the paired variable model was evaluated using repeated measures analysis of variance or Friedman’s test (for non-normally distributed data or ordinal data). Chi-square tests of independence were used for categorical variables (with Yates correction for cell counts below 10, checking Cochran’s conditions, and Fisher’s exact test). To determine the strength and direction of the relationship between variables, correlation analysis was performed using Pearson and/or Spearman correlation coefficients. In all calculations, the level of significance was set at *p* = 0.05.

## 4. Results

**FIGURE 1:**
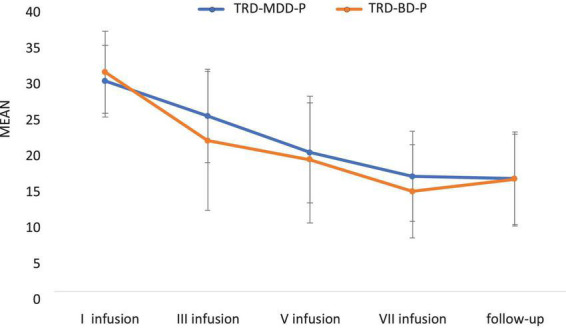
Means and standard errors for MADRS scores over the time of ketamine treatment in studied groups.

**FIGURE 2:**
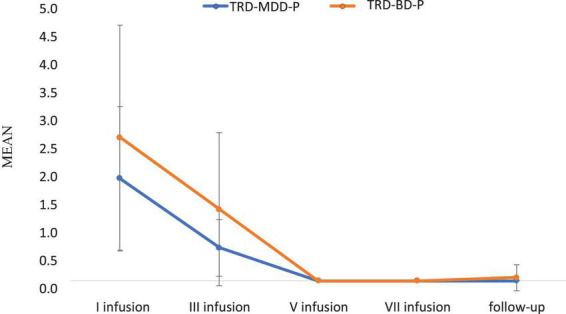
Means and standard errors for MADRS-item 10 (suicidal thoughts) scores over the time of ketamine treatment in studied groups.

**FIGURE 3:**
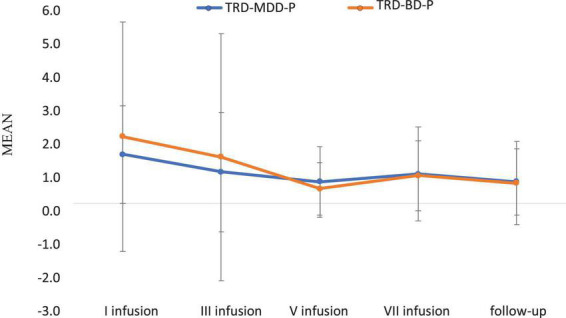
Means and standard errors for YMRS scores over the time of ketamine treatment in studied groups.

**FIGURE 4:**
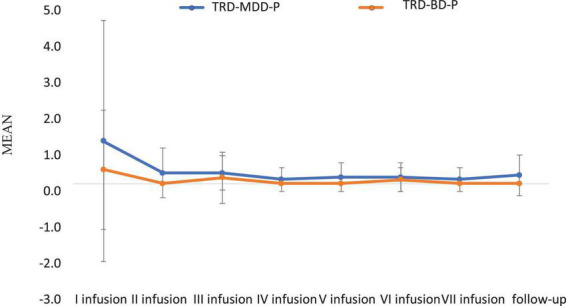
Means and standard errors for CADSS scores measured before the ketamine infusions in studied groups.

**FIGURE 5:**
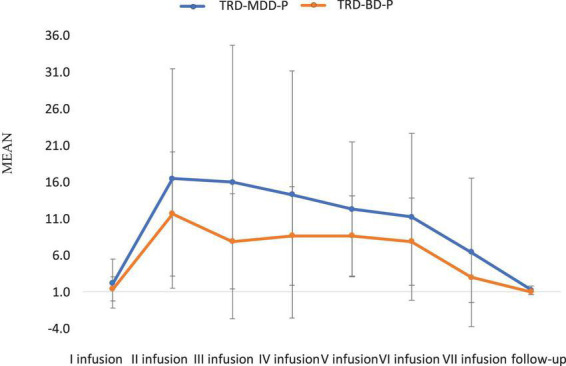
Means and standard errors for CADSS scores measured 30 min after the ketamine infusions in studied groups.

**FIGURE 6:**
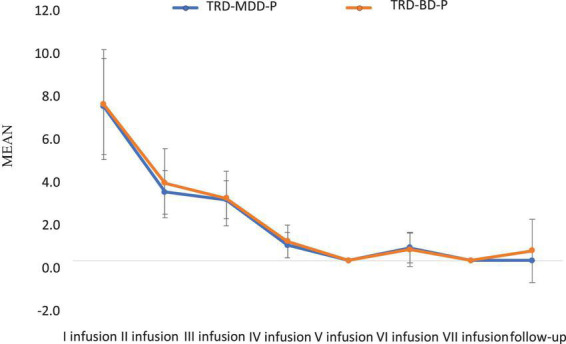
Means and standard errors for BPRS scores measured before the ketamine infusions in studied groups.

**FIGURE 7:**
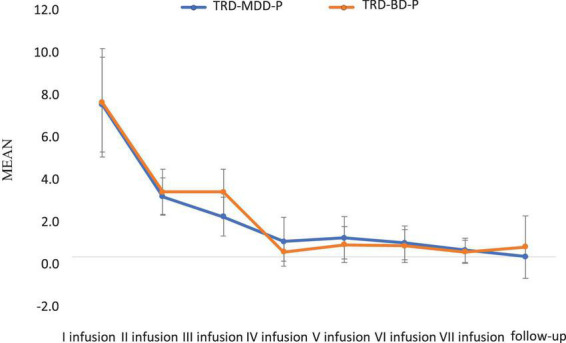
Means and standard errors for BPRS scores measured 30 min after the ketamine infusions in studied groups.

The demographic and clinical attributes of the study cohorts are depicted in Table 1. Figure 1 and Table 2 display the fluctuations in MADRS scores over time, which are consistent with other studies demonstrating clinical improvement during ketamine therapy (Kryst et al., 2020). Figure 2 and Table 3 exhibit item 10 from MADRS (suicidal ideation) and its temporal variations, indicating a reduction in suicidal thoughts, as corroborated by previous research on the subject (Abbar et al., 2022). Figure 3 and Table 4 demonstrate no significant changes in YMRS scores during ketamine therapy, suggesting that none of our patients exhibited an affective switch. Data on affective switch during ketamine therapy in the literature are limited and inconsistent, necessitating further investigation (Niciu et al., 2013; Allen et al., 2019; Wilkowska et al., 2020). We observed a decrease in dissociative symptoms in both groups (Figures 4, 5 and Tables 5, 6), as measured by CADSS before and 30 min after infusion. Similarly, we observed an improvement in psychotic symptoms, as measured by BPRS before and 30 min after ketamine infusion (Figures 6, 7 and Tables 7, 8), in both groups.

**TABLE 1:** Demographic and clinical variables.

	TRD-MDD-P (*n* = 17)	TRD-BD-P (*n* = 18)	*P*-value
Sex			0.4074^3^
Female	9 (52.9%)	12 (66.7%)	
Male	8 (47.1%)	6 (33.3%)	
Mean age in years (SD)	51.2 (11.1)	46.3 (17.4)	0.3266^1^
Mean body mass index (SD)	27.2 (6.2)	27.4 (6.5)	0.9438^1^
Mean number of depressive episodes (SD)	2.6 (2.2)	6.5 (3.6)	0.0008^2^
Mean duration of depressive episode in weeks (SD)	23.9 (19.5)	28.7 (32.1)	0.8301^2^
Bipolar disorder type I	0 (0.0%)	12 (66.7%)	<0.0001^3^
Bipolar disorder type II	0 (0.0%)	6 (33.3%)	0.0089^3^
Comorbidities			
Hypertension	7 (41.2%)	5 (27.8%)	0.4039^3^
Diabetes	1 (5.9%)	1 (5.6%)	0.9668^3^
Epilepsy	1 (5.9%)	3 (16.7%)	0.3162^3^
Hypercholesterolemia	3 (17.6%)	1 (5.6%)	0.2611^3^
Other	4 (23.5%)	6 (33.3%)	0.5211^3^
Co-existing treatment			
Tricyclic antidepressants			
Amitryptyline	2 (11.8%)	0 (0.0%)	0.1340^3^
Clomipramine	3 (17.6%)	0 (0.0%)	0.0623^3^
Selective serotonin reuptake inhibitors			
Fluvoxamine	0 (0.0%)	1 (5.6%)	0.3241^3^
Paroxetine	2 (11.8%)	2 (11.1%)	0.9516^3^
Fluoxetine	4 (23.5%)	4 (22.2%)	0.9267^3^
Sertraline	4 (23.5%)	4 (22.2%)	0.9267^3^
Citalopram	1 (5.9%)	1 (5.6%)	0.9668^3^
Escitalopram	2 (11.8%)	0 (0.0%)	0.1340^3^
Selective serotonine-noradrenaline reuptake inhibitors			
Venlafaxine	6 (35.3%)	5 (27.8%)	0.6321^3^
Duloxetine	2 (11.8%)	1 (5.6%)	0.5119^3^
Other antidepressants			
Mirtazapine	5 (29.4%)	2 (11.1%)	0.1761^3^
Mianserin	0 (0.0%)	1 (5.6%)	0.3241^3^
Trazodone	1 (5.9%)	0 (0.0%)	0.2965^3^
Bupropion	1 (5.9%)	2 (11.1%)	0.5808^3^
Vortioxetine	1 (5.9%)	0 (0.0%)	0.2965^3^
Antipsychotics			
Aripiprazole	1 (5.9%)	5 (27.8%)	0.0858^3^
Quetiapine	4 (23.5%)	8 (44.4%)	0.1926^3^
Olanzapine	2 (11.8%)	6 (33.3%)	0.1288^3^
Zuclopentixol	1 (5.9%)	0 (0.0%)	0.2965^3^
Risperidone	0 (0.0%)	1 (5.6%)	0.3241^3^
Mood stabilizers			
Lithium	1 (5.9%)	8 (44.4%)	0.0091^3^
Valproate	2 (11.8%)	6 (33.3%)	0.1288^3^
Lamotrigine	3 (17.6%)	7 (38.9%)	0.1644^3^

^1^t-Student; ^2^U Mann–Whitney; ^3^Chi-squared; SD, standard deviation; TRD-MDD-P, treatment resistant major psychotic depression; TRD-BD-P, treatment resistant bipolar psychotic depression.

**TABLE 2:** Comparative characteristics of the studied groups in terms of MADRS.

	TRD-MDD-P (*n* = 17)	TRD-BD-P (*n* = 18)	*P*-value
I infusion			0.6440^2^
SD	29.4 (5.0)	30.6 (5.7)	
Range	22.0–36.0	20.0–40.0	
Median (IQR)	29.0 (10.0)	32.5 (8.0)	
95% CI	[26.8; 31.9]	[27.7; 33.4]	
III infusion			0.2245^1^
SD	24.5 (6.5)	21.0 (9.7)	
Range	14.0–35.0	3.0–39.0	
Median (IQR)	27.0 (11.0)	20.0 (13.0)	
95% CI	[21.1; 27.8]	[16.2; 25.8]	
V infusion			0.7244^1^
SD	19.4 (7.0)	18.4 (8.9)	
Range	10.0–34.0	4.0–32.0	
Median (IQR)	19.0 (7.0)	17.5 (15.0)	
95% CI	[15.8; 22.9]	[14.0; 22.8]	
VII infusion			0.3365^1^
SD	16.1 (6.3)	13.9 (6.5)	
Range	8.0–29.0	4.0–25.0	
Median (IQR)	16.0 (7.0)	13.5 (8.0)	
95% CI	[12.8; 19.3]	[10.7; 17.2]	
Follow-up			0.8819^2^
SD	15.7 (6.6)	15.7 (6.3)	
Range	9.0–30.0	4.0–28.0	
Median (IQR)	14.0 (6.0)	14.0 (9.0)	
95% CI	[12.3; 19.1]	[12.5; 18.8]	

^1^t-Student; ^2^U Mann-Whitney; SD, standard deviation; IQR, interquartile range; TRD-MDD-P, treatment resistant major psychotic depression; TRD-BD-P, treatment resistant bipolar psychotic depression; MADRS, Montgomery Asberg Depression Scale.

**TABLE 3:** Comparative characteristics of the studied groups in terms of MADRS – item 10 (suicidal thoughts).

	TRD-MDD-P (*n* = 17)	TRD-BD-P (*n* = 18)	*P*-value
I infusion			0.3908^1^
SD	1.8 (1.3)	2.6 (2.0)	
Range	0.0–4.0	0.0–6.0	
Median (IQR)	1.0 (2.0)	3.5 (3.0)	
95% CI	[1.2; 2.5]	[1.6; 3.6]	
III infusion			0.2761^1^
SD	0.6 (0.5)	1.3 (1.4)	
Range	0.0–1.0	0.0–4.0	
Median (IQR)	1.0 (1.0)	1.0 (3.0)	
95% CI	[0.3; 0.8]	[0.6; 2.0]	
V infusion			0.9868^1^
SD	0.0 (0.0)	0.0 (0.0)	
Range	0.0–0.0	0.0–0.0	
Median (IQR)	0.0 (0.0)	0.0 (0.0)	
95% CI	[0.0; 0.0]	[0.0; 0.0]	
VII infusion			0.9868^1^
SD	0.0 (0.0)	0.0 (0.0)	
Range	0.0–0.0	0.0–0.0	
Median (IQR)	0.0 (0.0)	0.0 (0.0)	
95% CI	[0.0; 0.0]	[0.0; 0.0]	
Follow-up			0.7917^1^
SD	0.0 (0.0)	0.1 (0.2)	
Range	0.0–0.0	0.0–1.0	
Median (IQR)	0.0 (0.0)	0.0 (0.0)	
95% CI	[0.0; 0.0]	[−0.1; 0.2]	

^1^U Mann-Whitney; SD, standard deviation; IQR, interquartile range; TRD-MDD-P, treatment resistant major psychotic depression; TRD-BD-P, treatment resistant bipolar psychotic depression; MADRS, Montgomery Asberg Depression Scale.

**TABLE 4:** Comparative characteristics of the studied groups in terms of YMRS.

	TRD-MDD-P (*n* = 17)	TRD-BD-P (*n* = 18)	*P*-value
I infusion			0.8560^1^
SD	1.5 (1.5)	2.0 (3.4)	
Range	0.0–5.0	0.0–14.0	
Median (IQR)	2.0 (2.0)	0.5 (3.0)	
95% CI	[0.7; 2.2]	[0.3; 3.7]	
III infusion			0.9342^1^
SD	0.9 (1.8)	1.4 (3.7)	
Range	0.0–6.0	0.0–15.0	
Median (IQR)	0.0 (1.0)	0.0 (1.0)	
95% CI	[0.0; 1.9]	[−0.4; 3.2]	
V infusion			0.6799^1^
SD	0.6 (1.1)	0.4 (0.8)	
Range	0.0–3.0	0.0–2.0	
Median (IQR)	0.0 (1.0)	0.0 (1.0)	
95% CI	[0.1; 1.2]	[0.1; 0.8]	
VII infusion			0.8689^1^
SD	0.9 (1.4)	0.8 (1.0)	
Range	0.0–3.0	0.0–3.0	
Median (IQR)	0.0 (3.0)	0.0 (2.0)	
95% CI	[0.2; 1.6]	[0.3; 1.4]	
Follow-up			0.8173^1^
SD	0.6 (1.0)	0.6 (1.2)	
Range	0.0–3.0	0.0–5.0	
Median (IQR)	0.0 (1.0)	0.0 (1.0)	
95% CI	[0.1; 1.2]	[0.0; 1.2]	

^1^U Mann-Whitney; SD, standard deviation; IQR, interquartile range; TRD-MDD-P, treatment resistant major psychotic depression; TRD-BD-P, treatment resistant bipolar psychotic depression; YMRS, Young Mania Rating Scale.

**TABLE 5:** Comparative characteristics of the studied groups in terms of CADSS scores measured before the ketamine infusions.

	TRD-MDD-P (*n* = 17)	TRD-BD-P (*n* = 18)	*P*-value
I infusion			0.0892^1^
SD	1.2 (3.3)	0.4 (1.6)	
Range	0.0–14.0	0.0–7.0	
Median (IQR)	0.0 (1.0)	0.0 (0.0)	
95% CI	[−0.5; 2.9]	[−0.4; 1.2]	
II infusion			0.3818^1^
SD	0.3 (0.7)	0.0 (0.0)	
Range	0.0–2.0	0.0–0.0	
Median (IQR)	0.0 (0.0)	0.0 (0.0)	
95% CI	[−0.1; 0.6]	[0.0; 0.0]	
III infusion			0.2689^1^
SD	0.3 (0.5)	0.2 (0.7)	
Range	0.0–1.0	0.0–3.0	
Median (IQR)	0.0 (1.0)	0.0 (0.0)	
95% CI	[0.1; 0.5]	[−0.2; 0.5]	
IV infusion			0.5635^1^
SD	0.1 (0.3)	0.0 (0.0)	
Range	0.0–1.0	0.0–0.0	
Median (IQR)	0.0 (0.0)	0.0 (0.0)	
95% CI	[−0.1; 0.3]	[0.0; 0.0]	
V infusion			0.3818^1^
SD	0.2 (0.4)	0.0 (0.0)	
Range	0.0–1.0	0.0–0.0	
Median (IQR)	0.0 (0.0)	0.0 (0.0)	
95% CI	[0.0; 0.4]	[0.0; 0.0]	
VI infusion			0.7539^1^
SD	0.2 (0.4)	0.1 (0.3)	
Range	0.0–1.0	0.0–1.0	
Median (IQR)	0.0 (0.0)	0.0 (0.0)	
95% CI	[0.0; 0.4]	[0.0; 0.3]	
VII infusion			0.5635^1^
SD	0.1 (0.3)	0.0 (0.0)	
Range	0.0–1.0	0.0–0.0	
Median (IQR)	0.0 (0.0)	0.0 (0.0)	
95% CI	[−0.1; 0.3]	[0.0; 0.0]	
Follow-up			0.3818^1^
SD	0.2 (0.6)	0.0 (0.0)	
Range	0.0–2.0	0.0–0.0	
Median (IQR)	0.0 (0.0)	0.0 (0.0)	
95% CI	[−0.1; 0.5]	[0.0; 0.0]	

^1^U Mann-Whitney; SD, standard deviation; IQR, interquartile range; TRD-MDD-P, treatment resistant major psychotic depression; TRD-BD-P, treatment resistant bipolar psychotic depression; CADSS, Clinican Administered Dissociative States Scale.

**TABLE 6:** Comparative characteristics of the studied groups in terms of CADSS scores measured 30 min after the ketamine infusions.

	TRD-MDD-P (*n* = 17)	TRD-BD-P (*n* = 18)	*P*-value
I infusion			0.0892^1^
SD	1.2 (3.3)	0.4 (1.6)	
Range	0.0–14.0	0.0–7.0	
Median (IQR)	0.0 (1.0)	0.0 (0.0)	
95% CI	[−0.5; 2.9]	[−0.4; 1.2]	
II infusion			0.5415^1^
SD	15.5 (15.0)	10.7 (8.5)	
Range	0.0–49.0	0.0–33.0	
Median (IQR)	11.0 (18.0)	10.5 (12.0)	
95% CI	[7.8; 23.2]	[6.4; 14.9]	
III infusion			0.1607^1^
SD	15.0 (18.7)	6.9 (6.5)	
Range	0.0–65.0	0.0–22.0	
Median (IQR)	10.0 (10.0)	3.5 (9.0)	
95% CI	[5.4; 24.6]	[3.6; 10.1]	
IV infusion			0.6921^1^
SD	13.3 (16.9)	7.7 (6.7)	
Range	0.0–62.0	0.0–24.0	
Median (IQR)	8.0 (17.0)	7.0 (10.0)	
95% CI	[4.6; 22.0]	[4.3; 11.0]	
V infusion			0.4678^1^
SD	11.3 (9.2)	7.7 (5.5)	
Range	0.0–28.0	0.0–15.0	
Median (IQR)	9.0 (13.0)	9.5 (10.0)	
95% CI	[6.6; 16.0]	[4.9; 10.4]	
VI infusion			0.6921^1^
SD	10.2 (11.4)	6.9 (6.0)	
Range	0.0–35.0	0.0–17.0	
Median (IQR)	7.0 (16.0)	6.0 (11.0)	
95% CI	[4.4; 16.1]	[3.9; 9.9]	
VII infusion			1.0000^1^
SD	5.4 (10.2)	2.0 (3.4)	
Range	0.0–33.0	0.0–15.0	
Median (IQR)	1.0 (2.0)	2.0 (2.0)	
95% CI	[0.2; 10.6]	[0.3; 3.7]	
Follow-up			0.0892^1^
SD	0.2 (0.6)	0.0 (0.0)	
Range	0.0–2.0	0.0–0.0	
Median (IQR)	0.0 (0.0)	0.0 (0.0)	
95% CI	[−0.1; 0.5]	[0.0; 0.0]	

^1^U Mann-Whitney; SD, standard deviation; IQR, interquartile range; TRD-MDD-P, treatment resistant major psychotic depression; TRD-BD-P, treatment resistant bipolar psychotic depression; CADSS, Clinican Administered Dissociative States Scale.

**TABLE 7:** Comparative characteristics of the studied groups in terms of BPRS scores measured before the ketamine infusions.

	TRD-MDD-P (*n* = 17)	TRD-BD-P (*n* = 18)	*P*-value
I infusion			0.6440^1^
SD	7.2 (2.2)	7.3 (2.6)	
Range	3.0–11.0	0.0–11.0	
Median (IQR)	7.0 (3.0)	7.5 (3.0)	
95% CI	[6.0; 8.3]	[6.0; 8.6]	
II infusion			0.2548^1^
SD	3.2 (1.0)	3.6 (1.6)	
Range	1.0–5.0	0.0–6.0	
Median (IQR)	3.0 (1.0)	4.0 (2.0)	
95% CI	[2.7; 3.7]	[2.8; 4.4]	
III infusion			0.5974^1^
SD	2.8 (0.9)	2.9 (1.3)	
Range	1.0–4.0	0.0–4.0	
Median (IQR)	3.0 (1.0)	3.0 (2.0)	
95% CI	[2.4; 3.3]	[2.3; 3.5]	
IV infusion			0.5415^1^
SD	0.7 (0.6)	0.9 (0.8)	
Range	0.0–2.0	0.0–2.0	
Median (IQR)	1.0 (1.0)	1.0 (1.0)	
95% CI	[0.4; 1.0]	[0.5; 1.3]	
V infusion			0.9868^1^
SD	0.0 (0.0)	0.0 (0.0)	
Range	0.0–0.0	0.0–0.0	
Median (IQR)	0.0 (0.0)	0.0 (0.0)	
95% CI	[0.0; 0.0]	[0.0; 0.0]	
VI infusion			0.6440^1^
SD	0.6 (0.7)	0.5 (0.8)	
Range	0.0–2.0	0.0–3.0	
Median (IQR)	0.0 (1.0)	0.0 (1.0)	
95% CI	[0.2; 1.0]	[0.1; 0.9]	
VII infusion			0.9868^1^
SD	0.0 (0.0)	0.0 (0.0)	
Range	0.0–0.0	0.0–0.0	
Median (IQR)	0.0 (0.0)	0.0 (0.0)	
95% CI	[0.0; 0.0]	[0.0; 0.0]	
Follow-up			0.5860^1^
SD	0.0 (0.0)	0.4 (1.5)	
Range	0.0–0.0	0.0–6.0	
Median (IQR)	0.0 (0.0)	0.0 (0.0)	
95% CI	[0.0; 0.0]	[−0.3; 1.2]	

^1^U Mann-Whitney; SD, standard deviation; IQR, interquartile range; TRD-MDD-P, treatment resistant major psychotic depression; TRD-BD-P, treatment resistant bipolar psychotic depression; BPRS, Brief Psychiatric Rating Scale.

**TABLE 8:** Comparative characteristics of the studied groups in terms of BPRS scores measured 30 min after the ketamine infusions.

	TRD-MDD-P (*n* = 17)	TRD-BD-P (*n* = 18)	*P*-value
I infusion			0.6440^1^
SD	7.2 (2.2)	7.3 (2.6)	
Range	3.0–11.0	0.0–11.0	
Median (IQR)	7.0 (3.0)	7.5 (3.0)	
95% CI	[6.0; 8.3]	[6.0; 8.6]	
II infusion			0.4000^1^
SD	2.8 (0.9)	3.1 (1.1)	
Range	1.0–4.0	1.0–4.0	
Median (IQR)	3.0 (1.0)	3.0 (2.0)	
95% CI	[2.4; 3.3]	[2.5; 3.6]	
III infusion			0.0037^1^
SD	1.9 (0.9)	3.1 (1.1)	
Range	0.0–4.0	1.0–4.0	
Median (IQR)	2.0 (1.0)	3.0 (2.0)	
95% CI	[1.4; 2.4]	[2.5; 3.6]	
IV infusion			0.2834^1^
SD	0.7 (1.2)	0.2 (0.4)	
Range	0.0–4.0	0.0–1.0	
Median (IQR)	0.0 (1.0)	0.0 (0.0)	
95% CI	[0.1; 1.3]	[0.0; 0.4]	
V infusion			0.3062^1^
SD	0.9 (1.0)	0.6 (0.9)	
Range	0.0–3.0	0.0–3.0	
Median (IQR)	1.0 (1.0)	0.0 (1.0)	
95% CI	[0.4; 1.4]	[0.1; 1.0]	
VI infusion			0.5747^1^
SD	0.6 (0.8)	0.5 (0.8)	
Range	0.0–2.0	0.0–3.0	
Median (IQR)	0.0 (1.0)	0.0 (1.0)	
95% CI	[0.2; 1.1]	[0.1; 0.9]	
VII infusion			0.7539^1^
SD	0.3 (0.6)	0.2 (0.5)	
Range	0.0–2.0	0.0–2.0	
Median (IQR)	0.0 (0.0)	0.0 (0.0)	
95% CI	[0.0; 0.6]	[−0.1; 0.5]	
Follow-up			0.5860^1^
SD	0.0 (0.0)	0.4 (1.5)	
Range	0.0–0.0	0.0–6.0	
Median (IQR)	0.0 (0.0)	0.0 (0.0)	
95% CI	[0.0; 0.0]	[−0.3; 1.2]	

^1^U Mann-Whitney; SD, standard deviation; IQR, interquartile range; TRD-MDD-P, treatment resistant major psychotic depression; TRD-BD-P, treatment resistant bipolar psychotic depression; BPRS, Brief Psychiatric Rating Scale.

## 5. Discussion

This study provides support for the lack of exacerbation of psychotic symptoms in both unipolar and bipolar depression. In the main contribution of this paper is the inclusion of patients with unipolar and bipolar depression with current psychotic features, as previous literature has focused both on those with a current and past history of psychotic features.

Veraart et al. (2021) conducted a review of 482 article abstracts and included 9 articles that reported on the use of ketamine for treating patients with a history of psychosis or current psychotic symptoms. Five of these articles reported on the use of ketamine to treat patients with unipolar or bipolar depression or depression in schizoaffective disorder. The remaining 4 studies investigated the use of ketamine for treating patients with schizophrenia. All of the studies were either case reports or pilot studies, and the total number of participants was 41. The primary aim of ketamine administration in all studies except one was to relieve depressive symptoms. The one exception was a trial that investigated the effects of ketamine administration on negative symptoms in 6 patients with schizophrenia.

A secondary analysis was performed to investigate the influence of lifetime history of psychosis on the response of patients to ketamine in depression trials. They pooled data from three randomized, placebo-controlled crossover trials involving patients with a current depressive episode who received 0.5 mg/kg ketamine infusion over 40 min. Two of the trials included patients with bipolar disorder receiving lithium or valproate treatment, and the third trial included unmedicated patients. All patients were free of any other psychotropic medication, including antipsychotics. Of the 69 patients for whom information on history of psychosis was available, 2 had major depressive disorder with psychotic features and 10 had bipolar disorder with psychotic features in the past. Patients with a history of psychosis showed an improvement in depressive symptoms. While the antidepressant effects of ketamine were significant in both groups when compared to placebo, they appeared to be less robust in patients with a positive history of psychosis than in those without. Scores on the CADSS were significantly higher in patients with a history of psychosis, but only 40 min post-infusion and not at later time points. Scores on the BPRS-P did not significantly differ between the two groups. Overall, this analysis suggests that a single infusion of ketamine in patients with a history of psychosis has antidepressant effects without causing psychotic symptoms (Pennybaker et al., 2017).

In case reports by da Frota Ribeiro et al. (2016) the use of ketamine as an antidepressant for patients with current psychotic features was described. The first patient was a 52-year-old woman with a treatment resistant unipolar depression with psychotic symptoms. The second one was a 55-year-old woman with schizoaffective disorder who presented with depression, severe suicidal ideation and catatonia. Both patients were treated with a 0.5 mg/kg IV ketamine infusion over 40 min, experienced improvement in mood and the cessation of psychotic symptoms.

Ajub and Lacerda (2018) conducted a study on the effectiveness of esketamine in four patients with major depressive disorder, bipolar depressive disorder with mixed features, or schizoaffective disorder, all with psychotic depressive symptoms. One patient had comorbid social anxiety disorder, and another had current alcohol abuse or dependence. Esketamine was administered either intravenously or subcutaneously at a dosage of 0.5 mg/kg. Three of the patients showed significant improvement or complete remission of both depressive and psychotic symptoms, one patient did not show any improvement after three weekly esketamine administrations. Mild to intense side effects such as dissociative symptoms, nausea, vomiting and light-headedness were reported but remitted within 2 h after administration. There were no reported worsening of psychotic symptoms after esketamine administration in any of the four patients.

In our earlier study involving four patients with TRD-MDD-P who received ketamine as an adjunctive therapy, we demonstrated favorable safety and tolerance in terms of depressive and psychotic symptoms. We did not observe any exacerbation of psychotic symptoms during short- or long-term follow-up (Gałuszko-Wȩgielnik et al., 2023).

Despite the limited data, current literature indicates that short-term ketamine treatment may be a safe and effective option for patients with a history of psychosis or ongoing psychotic symptoms.

Major limitation in our report is small sample group size and short follow-up time. Furthermore, the study was conducted at a single site, and the observational design did not incorporate treatment blinding or a control group. Another constraint was that the CADSS evaluation was only conducted 30 min after each dosage, rather than at multiple time points. Consequently, we were unable to determine the exact timeline for the peak of dissociative symptoms or their resolution.

Due to the limited data available for the presented study population and small sample sizes, the findings should be approached with caution and considered a preliminary report in the field.

## Data availability statement

The original contributions presented in this study are included in the article/supplementary material, further inquiries can be directed to the corresponding author.

## Ethics statement

The studies involving human participants were reviewed and approved by the Independent Bioethics Committee for Scientific Research at Medical University of Gdansk, Poland (the research protocols: NKBBN/172/2017; 172-674/2019). The patients/participants provided their written informed consent to participate in this study.

## Author contributions

MG-W, WC, and KJ-W: conceptualization and writing–original draft preparation. MG-W, WC, MW, and KJ-W: methodology. MP and KJ-W: writing–review and editing. All authors have read and agreed to the published version of the manuscript.
